# Variable levels of drift in tunicate cardiopharyngeal gene regulatory elements

**DOI:** 10.1186/s13227-019-0137-2

**Published:** 2019-10-11

**Authors:** William Colgan, Alexis Leanza, Ariel Hwang, Melissa B. DeBiasse, Isabel Llosa, Daniel Rodrigues, Hriju Adhikari, Guillermo Barreto Corona, Saskia Bock, Amanda Carillo-Perez, Meagan Currie, Simone Darkoa-Larbi, Daniel Dellal, Hanna Gutow, Pascha Hokama, Emily Kibby, Noah Linhart, Sophia Moody, Allison Naganuma, Diep Nguyen, Ryan Stanton, Sierra Stark, Cameron Tumey, Anthony Velleca, Joseph F. Ryan, Brad Davidson

**Affiliations:** 1 Broad Institute, Cambridge, USA; 2 Swarthmore College, Swarthmore, USA; 3 Thomas Jefferson University Sidney Kimmel Medical College, Philadelphia, USA; 4 University of North Carolina, Chapel Hill, USA; 5 Whitney Laboratory for Marine Bioscience, St. Augustine, USA; 6 University of Florida, Gainesville, USA; 7 Icahn School of Medicine at Mount Sinai, New York, USA; 8 Perelman School of Medicine, Philadelphia, USA; 9 University of Colorado Boulder, Boulder, USA; 10 Duke University School of Medicine, Durham, USA; 11 Rothman Institute, Philadelphia, USA; 12 University of Pittsburgh, Pittsburgh, USA; 13 University of California San Francisco, San Francisco, USA; 14 Memorial Sloan Kettering Cancer Center, New York, USA

**Keywords:** Gene regulatory networks, Developmental systems drift, Tunicates, Heart development, Selective constraints

## Abstract

**Background:**

Mutations in gene regulatory networks often lead to genetic divergence without impacting gene expression or developmental patterning. The rules governing this process of developmental systems drift, including the variable impact of selective constraints on different nodes in a gene regulatory network, remain poorly delineated.

**Results:**

Here we examine developmental systems drift within the cardiopharyngeal gene regulatory networks of two tunicate species, *Corella inflata* and *Ciona robusta.* Cross-species analysis of regulatory elements suggests that *trans*-regulatory architecture is largely conserved between these highly divergent species. In contrast, *cis*-regulatory elements within this network exhibit distinct levels of conservation. In particular, while most of the regulatory elements we analyzed showed extensive rearrangements of functional binding sites, the enhancer for the cardiopharyngeal transcription factor *FoxF* is remarkably well-conserved. Even minor alterations in spacing between binding sites lead to loss of *FoxF* enhancer function, suggesting that bound *trans*-factors form position-dependent complexes.

**Conclusions:**

Our findings reveal heterogeneous levels of divergence across cardiopharyngeal *cis*-regulatory elements. These distinct levels of divergence presumably reflect constraints that are not clearly associated with gene function or position within the regulatory network. Thus, levels of *cis*-regulatory divergence or drift appear to be governed by distinct structural constraints that will be difficult to predict based on network architecture.

## Background

The gene regulatory networks (GRNs) that orchestrate development are largely composed of *trans*-regulatory factors (i.e., transcription factors) and *cis*-regulatory elements (i.e., enhancers and silencers) [1]. Connections within these networks are dictated by transcription factor binding sites within each regulatory element [1–3]. Mutations that alter binding site composition are a major driver of developmental changes underlying evolutionary shifts in phenotype [4–9]. However, mutations can accumulate in *cis*-regulatory elements without altering gene network function, contributing to developmental systems drift [10–12]. Drift can also occur in *trans* due to mutations that impact the expression or coding sequence of upstream transcription factors (as defined in relation to a specific target gene) [5]. In general, the organization of binding motifs within *cis*-regulatory elements is loosely constrained. This structural flexibility presumably reflects independent, non-cooperative binding of upstream transcription factors [3, 13, 14]. However, within a limited subset of regulatory elements, the binding site organization is more tightly constrained. This structural rigidity presumably reflects cooperative, position-specific interactions between bound transcription factors and associated co-factors [14–19]. The prevalence and nature of such cooperative binding interactions and the resulting impact on drift are outstanding questions in evolutionary developmental biology [3].

Although developmental systems drift in GRNs appears to be a common phenomenon in metazoan evolution, it can be difficult to study due to the requirement for rigorous cross-species analysis within well-characterized networks [11, 12, 20, 21]. Cross-species assays are used to determine the intelligibility of characterized *cis*-regulatory elements between two species and thus evaluate hypotheses regarding the amount of drift. Mutual intelligibility of a *cis*-regulatory element suggests that only *cis* drift has occurred [22–24]. In contrast, partial or complete loss of intelligibility indicates that *trans* drift has occurred [10, 25, 26]. It should be noted that results from cross-species analysis are not definitive. Alterations in GRN structure may be associated with shifts in temporal or spatial expression that are difficult to detect either because they are subtle or because available techniques (such as reporter assays) do not accurately reflect endogenous expression. Thus, in general, experimental evidence for developmental systems drift does not rule out a role for selection in driving observed shifts in GRN architecture.

**Fig. 1:**
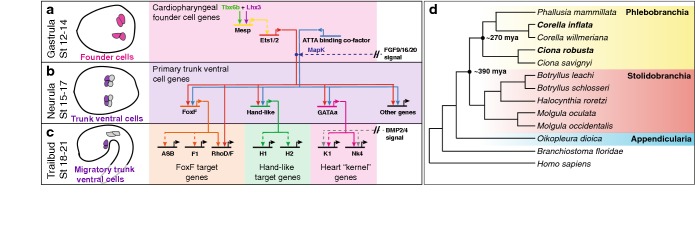
*Ciona robusta* cardiopharyngeal gene regulatory network and tunicate phylogeny. **a**–**c** Regulatory network diagrams for cardiopharyngeal founder lineage cells during three embryonic stages. Schematics on the left indicate stage and cell lineage. Background colors delineate discrete regulatory modules. Solid lines indicate regulatory connections supported by functional enhancer analysis, while dashed lines indicate regulatory connections supported by expression data. Circles represent signal dependent activation and double slanted lines represent signal transduction. **a** Initial specification of the cardiopharyngeal founder cells (pink) through exclusive up-regulation of *Mesp* and subsequent expression of *Ets1/2*. **b** Signal-dependent regulation of early trunk ventral cell genes by Ets1/2 and an unknown ATTA-binding co-factor. Ets1/2 activation in the TVCs is dependent on FGF9/16/20 signaling transduced by the MapK pathway. **c** Presumptive modules differentially regulated by FoxF, Hand-like, or GATAa. FoxF is portrayed as the primary regulator of TVC migration, while GATAa regulates a highly conserved heart “kernel” in conjunction with BMP2/4 signaling. F1, H1, H2, and K1 represent hypothetical target genes. Figure is based on Woznica et al. and Cota et al. [41, 42]. **d** Simplified tunicate phylogeny based on DeBiasse et al. (in prep), that is congruent with Delsuc et al. [43]. Background colors represent sub-clades, *Phlebobranchia* (yellow), *Stolidobranchia* (red), or *Appendicularia* (blue)

Tunicates, or urochordates, are a powerful system for studying developmental systems drift (Fig. 1). They are closely related to vertebrates but diverged prior to vertebrate genome duplications, so they have a single copy of many important developmental genes [27, 28]. Tunicates also have relatively compact genomes, enabling easy identification of *cis*-regulatory elements through phylogenetic footprinting or detection of clustered binding motifs [29–32]. In addition, some tunicate species can be electroporated *en masse,* enabling high-throughput testing of *cis*-regulatory elements with transgenic reporters [33]. These techniques have been successfully employed to intensively characterize developmental gene regulatory networks in *Ciona robusta* (formerly known as *Ciona intestinalis*, type A), including the network underlying heart and pharyngeal development (Fig. 1a–c). Furthermore, tunicate embryos employ similar, deeply conserved patterning mechanisms for early development. Remarkably, species in two major tunicate clades, *Phlebobranchia* and *Stolidobranchia*, have nearly identical embryonic fate maps and employ similar programs for specification and morphogenesis, despite having diverged ~ 390 million years ago (Fig. 1d) [10, 34–36]. These similarities in developmental patterning are even more striking when the extreme rate of genomic divergence between tunicate species is taken into consideration [37–40]. The unique combination of stringent developmental conservation and extreme genomic divergence makes the tunicates a powerful model for revealing the constraints that shape adaptation and developmental systems drift [37].

Previous studies of tunicate developmental systems drift have focused on comparisons to the relatively well-characterized regulatory networks underlying embryonic development in *C. robusta* [44]. For some genes, including the key developmental transcription factor *Otx*, conservation of the *trans*-regulatory environment promotes conserved expression patterns and mutual intelligibility in cross-species analysis despite extensive binding site rearrangements within *cis*-regulatory elements [24, 45]. In other cases, expression is conserved despite divergence of the *trans*-regulatory factors and associated *cis*-regulatory elements, leading to loss of cross-species intelligibility [26]. Drift in *trans*-factors is also indicated by species-specific deployment of distinct signaling pathways in otherwise conserved developmental programs, including the program driving muscle progenitor lineage induction [46, 47]. These findings align with the hypothesis that the extreme genomic divergence between tunicate species has resulted in profound levels of drift within developmental GRNs [37].

Extensive characterization of the *C. robusta* cardiopharyngeal GRN makes it an attractive model for comparative studies examining developmental systems drift (Fig. 1a–c) [42, 48, 49]. The heart in *C. robusta* can be traced back to two blastomeres (the B7.5 cells, also termed cardiopharyngeal founder cells) which express the bHLH transcription factor *Mesp* (Fig. 1a) [50–52]. Founder cell-specific expression of *Mesp* is mediated by two upstream transcription factors: a T-Box family transcription factor, TBX6b, and a LIM homeobox family transcription factor, LHX3, which are expressed in overlapping maternally specified domains [51, 53, 54]. During gastrulation, the founder cells divide once, forming a pair of cells on each side of the embryo, and express the transcription factor *Ets1/2* (Fig. 1a). The four resulting cells then divide asymmetrically, creating two distinct cell lineages: the anterior tail muscle cells (ATMs) and the trunk ventral cells (TVCs). The TVCs are bi-potential progenitors, giving rise to pharyngeal muscle and cardiac lineages (Fig. 1b). TVC specification is dictated by fibroblast growth factor (FGF)/Map Kinase (MapK)-dependent activation of Ets1/2 [55–57]. Ets1/2 in conjunction with an unknown ATTA-binding co-factor then upregulates a set of 218 primary genes which include the conserved cardiac transcription factors *FoxF, Hand*-*like,* and *GATAa* (Fig. 1b) [41, 58, 59]. These three transcription factors are thought to regulate distinct modules in the *C. robusta* cardiopharyngeal GRN (Fig. 1c) [42, 60–63].

Comparative analysis of the *C. robusta* cardiopharyngeal GRN has been initiated in two species, *Ciona savignyi* and *Molgula occidentalis.* Regulatory elements and upstream *trans*-factors appear to be highly conserved in *C. robusta* and *C. savignyi* despite ~ 100 million years of rapid genomic divergence [29, 64]. In *M. occidentalis* and *C. robusta,* which diverged ~ 390 million years ago, cardiopharyngeal founder lineages still exhibit nearly identical patterns of cell division and transcription factor expression [10]. However, there have been partial or complete losses of intelligibility between cardiopharyngeal *cis*-regulatory elements in these two species, indicating that significant developmental systems drift has occurred both in *cis* and in *trans* [10].

To explore how evolutionary constraints influence drift in developmental programs, we have begun comparative studies of the cardiopharyngeal GRN in *Corella inflata,* a phlebobranch that diverged from *C. robusta *~ 270 million years ago (Fig. 1d) (DeBiasse et al. 2019, in prep) [43]. *C. inflata* is experimentally tractable, as synchronized *C. inflata* embryos can be electroporated *en masse* to test reporter constructs, and we recently sequenced its genome and transcriptome (DeBiasse et al. 2019, in prep). We used this genome to characterize enhancers for key genes in the cardiopharyngeal GRN, including *Mesp, FoxF,* and *Hand*-*like.* We show that the *trans*-regulatory architecture of the cardiopharyngeal GRN is largely conserved between *C. robusta* and *C. inflata,* but *cis*-regulatory elements within this GRN exhibit different levels of conservation. These differences correspond to different structural and functional constraints.

## Results

### *C. inflata* and *C. robusta* share a conserved TVC specification program

**Fig. 2:**
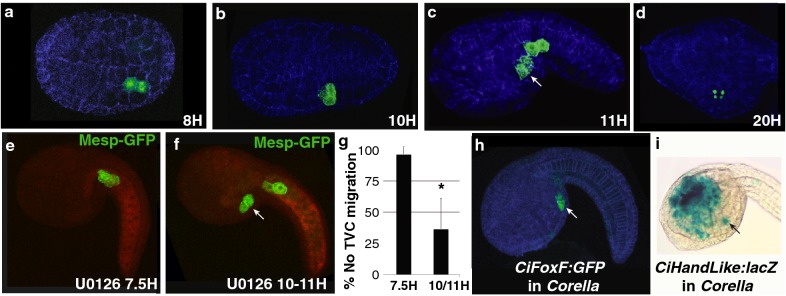
Conserved founder cell lineage behavior and TVC induction in *Corella* embryos. **a**–**c** Representative *Corella* embryos expressing *Cirobu.Mesp* −*1916:Esconsin*-*3XGFP* in presumptive founder lineage cells. Note labeling of mitotic spindle in 8H embryo (**a**). Hours post-fertilization indicated at the lower right of each panel. **d** Representative *Corella* embryo expressing *Cirobu.Mesp* −*1916:H2B:GFP* to track founder lineage cell divisions in later stages. **e**, **f** Transgenic *Cirobu.Mesp 1916:GFP Corella* embryos treated with the Map Kinase inhibitor U0126 at 7.5 HPF, **e** immediately prior to founder cell division or **f** ~ 2 h after division at 10-11 HPF. Arrow points to migrated TVCs. **g** Summary of results for U0126 treatments. Data spans 6 trials, *N* > 70 for each condition, Student’s *T* test, *p* value < 0.0005. Note that the levels of migration defects in the 10-11HPF treatment samples were similar to basal levels seen in untreated, transgenic embryos (data not shown). **h**, **i** Representative embryos illustrating TVC expression for the *Cirobu.FoxF*-3052:*GFP* and *Cirobu.Hand*-*like*-*2954/*− *445:*− *296:lacZ* reporters

**Fig. 3:**
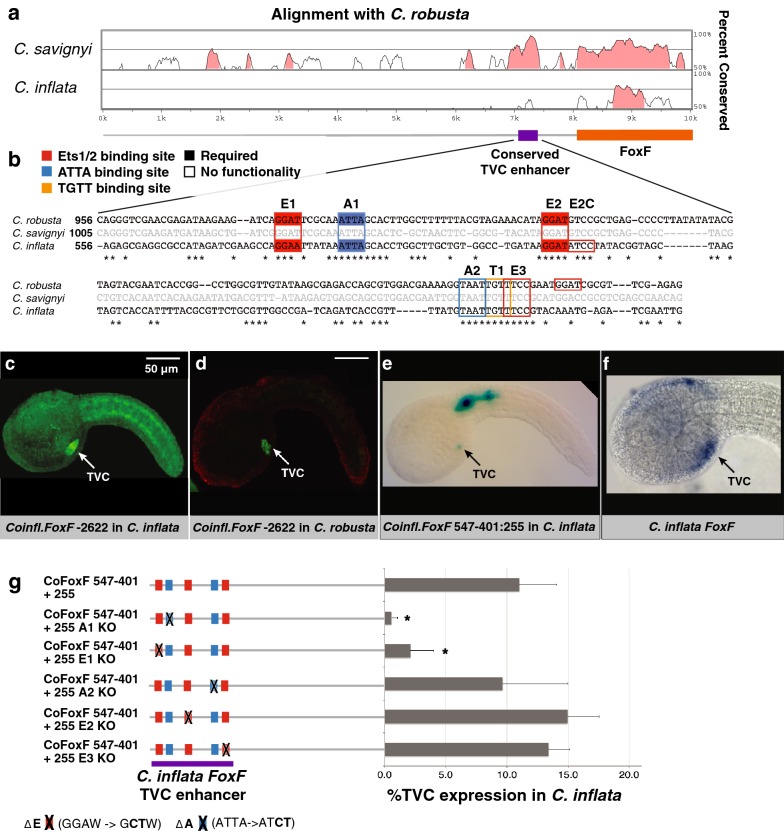
Characterization of the *C. inflata FoxF* TVC enhancer. **a** mVISTA alignments depict sequence conservation between *C. robusta* and *C. savignyi* and between *C. robusta* and *C. inflata* for the *FoxF* gene and 5′ intergenic region (LAGAN alignment, conservation across 100 bp window, conservation > 70% highlighted). There is increased conservation associated with the *FoxF* coding region (orange) and conserved TVC enhancer (purple). **b** ClustalW alignment of the 183 bp conserved TVC enhancer with Ets1/2 (red), ATTA (blue), and TGTT (orange)-binding motifs highlighted. Dark-shaded-binding motifs were required for reporter expression and boxed-binding motifs exhibited no functionality. *C. robusta FoxF-*binding motif knockout data come from Beh et al. and Woznica et al. [41, 58]. **c**–**e** Representative embryos showing the activity of *Coinfl.FoxF* −2622 *GFP* reporter constructs in *C. inflata* and *C. robusta* (arrows indicate expression in TVCs, and scale bar is 50 μm). **f** Representative *C. inflata* mid-tailbud stage embryo displaying expression of *Coinf.FoxF* in TVCs (arrow) and epidermis. **g** Effect of Ets1/2 and ATTA-binding motif knockouts (Δ) on reporter expression driven by the *C. inflata* 146 bp minimal TVC enhancer fused to a 255 bp basal promoter (*Coinfl.FoxF* −547/−401::−255). Names of binding motifs correspond to the names in **b**. *LacZ* reporter constructs are diagramed on the left with X indicating a binding motif knockout. The graph depicts %TVC expression in *C. inflata* (number of trials ≥ 2, total *N* ≥ 150, and error bars indicate standard deviation). Significance relative to *Coinfl.FoxF* −547/−401::−255 was determined with a Student’s *t* test, *p* < 0.05 indicated by *

To initiate our analysis of the *Corella* cardiopharyngeal GRN, we tested the activity of a characterized *C. robusta* reporter construct for the heart founder lineage transcription factor, *Mesp* (*Cirobu.Mesp*-*1916:Ensconsin:3XGFP)* [56]. Fortunately, electroporation protocols developed for* C. robusta *[30] were also effective for *C. inflata* embryos. As observed in *Ciona,* the *Cirobu.Mesp* enhancer drove robust activity in *Corella* B7.5 founder lineage cells, including both TVC and ATM lineages. The *Ensconsin:GFP* reporter labels microtubules [56, 65], allowing us to deploy this construct to track founder cell lineage position and division in developing *C. inflata* embryos. As seen previously in both *Molgulid* and *Cionid* species, bilateral pairs of *C. inflata* heart founder cells divide asymmetrically at the early neurula stage (~ 8HPF) to produce the heart progenitor and anterior tail muscle lineages (Fig. 2a, b). Further analysis will be required to determine if this division is unequal and whether differential induction involves receptor localization as characterized in *C. robusta* [57]. During tailbud stages, *C. inflata* heart progenitors undergo a conserved anterior migration along the epidermis into the ventral trunk region (Fig. 2c), where they undergo an unequal cleavage to form smaller medial and larger lateral daughters (Fig. 2d). Whether this represents an asymmetric division to produce pharyngeal muscle and heart precursors as seen in *C. robusta* will require further analysis [62]. We also used the *Cirobu.Mesp* reporter to examine whether TVC specification (as marked by anterior migration) is dependent on FGF/MapK signaling. As seen previously in both *C. robusta* and *Molgula occidentalis* embryos, treatment with the MEK inhibitor U0126 just prior to B7.5 founder cell division (late gastrula stage) blocked induction of the heart progenitor lineage (as indicated by lack of TVC migration, Fig. 2e, g), while treatment at a later time point had no effect (Fig. 2f, g) [10, 55]. We also began to examine conservation of the heart gene network downstream of FGF-dependent induction. In *C. robusta*, a small group of transcription factors including *FoxF*, *Hand*-*like,* and *GATAa* are upregulated directly downstream of FGF/MapK induction (Fig. 1) [41]. Through in situ hybridization in tailbud stage embryos, we found that *C. inflata FoxF* is expressed in the trunk epidermis and TVCs, mirroring similar expression in *C. robusta* embryos at this stage (Fig. 3f). This initial analysis indicates that the program for trunk ventral cell specification and migration in *C. inflata* and *C. robusta* embryos has been conserved.

### *C. robusta* cardiac gene enhancers drive TVC reporter expression in *C. inflata*

To further explore developmental systems drift in the cardiopharyngeal gene regulatory network, we began to perform cross-species testing of regulatory elements. Since *C. inflata* and *C. robusta* shared a common ancestor more recently than *C. robusta* and *M. occidentalis* (Fig. 1d) [43], we hypothesized that there would be conservation in the *trans*-regulatory architecture despite divergence of *cis*-regulatory elements. Based on this hypothesis, we expected the *C. inflata* and *C. robusta* cardiopharyngeal GRN enhancers to display mutual intelligibility in cross-species testing but not to align or exhibit similar binding site arrangements. Alternatively, it is possible that both *cis*-regulatory elements and *trans*-regulatory architecture have been conserved, as seen in comparisons between *C. savignyi* and *C. robusta* [29, 41, 50, 58], or that there has been divergence of both the *cis*-regulatory elements and *trans*-regulatory architecture, as seen in comparisons between *M. occidentalis* and *C. robusta* [10]. To begin exploring these hypotheses, we tested two well-characterized *C. robusta* TVC enhancers, *Cirobu.FoxF*-3052:*GFP* and *Cirobu.Hand-Like*-*2954*/−445:−296*:lacZ,* in *Corella* embryos. In *C. robusta,* both of these enhancer elements mediate TVC expression immediately after TVC induction and are co-regulated by Ets1/2 and an ATTA-binding co-factor [41, 58]. As seen with the *Cirobu.Mesp*-*1916* enhancer (Fig. 2a–f), both these reporters recapitulated characterized *Ciona* expression patterns in transgenic *Corella* embryos. The *FoxF* reporter drove expression in the TVCs and trunk epidermis (Fig. 2h) and the *Hand*-*like* reporter drove expression in the TVCs and trunk endoderm along with weak expression in the ATM lineage (Fig. 2i). The cross-species intelligibility of these three reporters indicates that TVC specification and migration in *Corella* and *Ciona* embryos rely on a conserved set of upstream *trans*-factors.

### The *FoxF* TVC enhancer is highly conserved between *C. inflata* and *C. robusta*

To further explore drift of the *FoxF*-regulatory element, we attempted to identify a candidate orthologous enhancer in *Corella* using mVISTA multi-sequence alignment [66]. This alignment revealed a small region of sequence conservation in *C. inflata* at the position of the previously characterized *C. robusta FoxF* TVC enhancer (Fig. 3a) [58]. Strikingly, this 183 bp region contained a set of three conserved Ets1/2 and two conserved ATTA-binding motifs that precisely matched the number, spacing, and arrangement of the characterized binding sites in the orthologous *Ciona FoxF* enhancer, while intervening DNA was poorly conserved (Fig. 3b). Reporter constructs containing this conserved element in *C. inflata* were able to drive TVC-specific expression in both *C. inflata* (Fig. 3c) and *C. robusta* (Fig. 3d). Thus, cross-species testing demonstrated mutual intelligibility of a remarkably well-conserved *FoxF* TVC enhancer (Figs. 2h, 3c, d).

To further evaluate whether the conserved region upstream of *Corella FoxF* represented a functionally constrained regulatory element, we cloned a 146 bp fragment containing the full set of conserved binding motifs. We then fused this minimal region to a 255 bp basal promoter that had no independent reporter expression (data not shown). The resulting construct (*Coinfl.FoxF* −547/−401::−255) drove reporter expression in *Corella* B7.5 lineage cells, including the TVCs and ATM precursors (Fig. 3e, g). We then individually knocked out the five conserved binding motifs in this minimal element through site-directed mutagenesis and visualized reporter expression in *C. inflata* embryos. While the disruption of the first Ets1/2 (E1) or first ATTA (A1) binding motifs significantly reduced TVC reporter expression, knocking out the other binding motifs had no discernible impact (Fig. 3g). These results mirrored the results from a similar analysis of the *C. robusta FoxF* TVC enhancer [41, 58] with the exception of the second Ets1/2 (E2)-binding motif which was required in the *C. robusta* enhancer (Fig. 3g). This apparent divergence in enhancer structure may reflect the presence of a third (presumably supplemental) Ets1/2-binding motif in *C. inflata* immediately adjacent to the second Ets1/2 motif (E2C), potentially creating redundancy. These results suggest that selection has stringently constrained *FoxF* TVC enhancer structure, preventing any major shifts in the order, number, or spacing of binding sites over nearly 300 million years of rapid genomic divergence between *C. robusta* and *C. inflata*.

### Differential divergence of the *Hand*-*like* vs. *FoxF* TVC enhancer elements

To determine if the rigorous conservation of the *FoxF* TVC enhancer was unique or reflected generally high levels of constraint in the cardiopharyngeal GRN, we characterized the *C. inflata* TVC enhancer for *Hand*-*like*. *Hand*-*like* and *FoxF* occupy very similar positions in the *C. robusta* cardiopharyngeal GRN [42]. Both these genes are expressed shortly after TVC induction. They are both regulated by Ets1/2 and an ATTA-binding co-factor and they encode key transcription factors for TVC progenitor fate (Fig. 1b). Based on the proposition that the hierarchical position of a gene within a GRN correlates with the level of selective constraint on its regulatory elements [4], we hypothesized that *Hand*-*like* and *FoxF-*regulatory elements would exhibit a similar level of conservation.

Sequence alignments did not reveal a conserved region in *C. inflata* associated with the characterized *Hand*-*like* TVC enhancer in *C. robusta* (Additional file 1: Figure S1A) [66]. However, this analysis did not exclude the presence of a conserved enhancer that may have shifted position relative to the *Hand*-*like* gene and thus failed to align globally. We, therefore, searched more broadly for the *C. inflata Hand*-*like* TVC enhancer based on binding motif clustering and organization (see methods for further details). This approach identified two strong candidate elements in the 5ʹ intergenic region (Additional file 1: Figure S1B). The distal element (prediction 1) was located 1737–1587 bp upstream of the gene, in a similar position to the previously characterized *C. robusta* enhancer. The proximal element (prediction 2) was located 1048–898 bp upstream of the gene. Both predicted elements contained Ets1/2 and ATTA-binding motifs and exhibited some structural similarity to the previously characterized TVC enhancer of *C. robusta Hand*-*like* (Additional file 1: Figure S1B) [41].

**Fig. 4:**
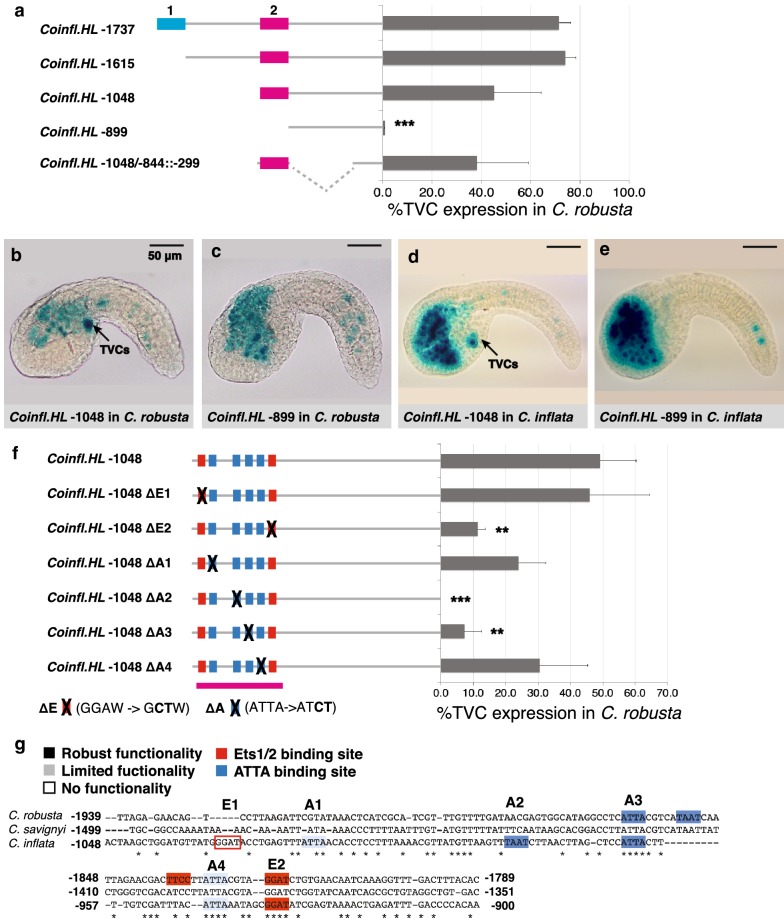
Characterization of the *C. inflata Hand*-*like* TVC enhancer. **a** Minimization of the *C. inflata Hand*-*like* (*HL*) upstream genomic fragment to test two predicted enhancers. *LacZ* reporter constructs are diagramed on the left. The graph depicts %TVC expression in *C. robusta* (number of trials ≥ 2, total *N* ≥ 75, and error bars indicate standard deviation). Significance relative to *Coinfl.HL* −1737 was determined with a Student *t* test (*p* < 0.001 indicated by ***). The second predicted enhancer is both necessary and sufficient for reporter expression in the TVCs. **b**–**e** Representative embryos showing the expression of *LacZ* reporter constructs that contain the second predicted enhancer (*Coinfl.HL* −1048) or lack the second predicted enhancer (*Coinfl.HL* −899) in both *C. robusta* and *C. inflata* (arrows indicate expression in TVCs, and scale bar is 50 μm). **f** Effect of Ets1/2 and ATTA-binding motif knockouts (Δ) on the expression of a *C. inflata Hand*-like::*LacZ* reporter construct containing a 1048 bp upstream genomic fragment (*Coinfl.HL* −1048). Names of binding motifs correspond to the names in panel B. *LacZ* reporter constructs are diagramed on the left with X indicating a binding motif knockout. The graph depicts %TVC expression in *C. robusta* (number of trials ≥ 2, total *N* ≥ 25, and error bars indicate standard deviation). Significance relative to *Coinfl.HL* −1048 was determined with a Student *t* test (*p* < 0.01 indicated by ** and *p* < .001 indicated by ***). **g** Comparison of *Hand*-*like* TVC enhancer structure in *C. robusta* and *C. inflata*. Darkly shaded binding motifs were required for reporter expression. Lightly shaded binding motifs exhibited ‘limited” functionality as assessed by mutagenesis of multiple sites in the minimal *Cirobu.FoxF* enhancer [41] or by a non-significant reduction in reporter expression following mutagenesis (this study). Boxed binding motifs exhibited no functionality. *C. robusta Hand*-*like* binding motif knockout data comes from Woznica et al. [41]

We tested these computational predictions through sequential minimization of the *C. inflata Hand*-*like* 5′ intergenic region using *LacZ* reporter constructs (Fig. 4a). The full-length construct (*Coinfl.HL* −1737) containing both candidate elements had strong TVC expression in *C. robusta,* demonstrating that the *Hand*-*like* TVC enhancer is intelligible by *C. robusta*. We employed *C. robusta* for further minimization experiments, because this species is more readily available than *C. inflata*. Deletions that removed the first candidate *cis*-regulatory element (*Coinfl.HL* −1615) or the region between the candidate *cis*-regulatory elements (*Coinfl.HL* −1048) did not affect TVC reporter expression (Fig. 4a, b), but removing the second candidate *cis*-regulatory element (*Coinfl.HL* −899) eliminated TVC reporter expression (Fig. 4a, c). A minimal 208 bp region encompassing the second candidate *cis*-regulatory element fused to a 299 bp basal promoter (*Coinfl.HL* −1048/−844::−299) drove strong TVC expression along with some ectopic expression in the mesenchyme, a hotspot for ectopic reporter expression [67], demonstrating that this region is both necessary and sufficient for *Hand*-*like* TVC expression (Fig. 4a). *Coinfl.HL* −1048 had strong TVC reporter expression (Fig. 4b) and *Coinfl.HL* −899 had no TVC reporter expression (Fig. 4c). Similar results were obtained in *C. inflata* (Fig. 4d, e). Thus, the *Hand*-*like* TVC enhancer is mutually intelligible in cross-species assays (Figs. 2i, 4c) while exhibiting substantially more divergence in binding motif organization in comparison with the *FoxF* TVC enhancer.

We next began to functionally characterize the binding sites in the *C. inflata Hand*-*like* TVC enhancer through site-directed mutagenesis (Fig. 4f). This enhancer contains two Ets1/2 and four ATTA-binding motifs (Fig. 4g). Knocking out the second or third ATTA motif (A2, A3) or the second Ets1/2 motif (E2) significantly reduced TVC reporter expression, while knocking out the remaining motifs did not significantly alter TVC reporter expression (Fig. 4f). In contrast, published mutational analysis of the *C. robusta Hand*-*like* element indicated that both Ets sites along with the first and second ATTA sites were required for full reporter activity (dark shading indicates functionally required binding motifs, Fig. 4f) [41]. In summary, our analysis indicates that *trans*-regulation of *Hand*-*like* expression in the TVCs by Ets1/2 and an ATTA-binding co-factor has been conserved between these two species, while the *cis*-regulatory element has undergone substantial divergence, including changes in the number, order, orientation, and spacing of binding motifs. Thus, the *cis*-regulatory elements for *FoxF* and *Hand*-*like* appear to have experienced distinct levels of functional constraint, despite occupying similar positions in the cardiopharyngeal GRN.

### FoxF functions upstream of *Hand-like* in the cardiopharyngeal GRN

When we aligned the *FoxF* and *Hand*-*like* TVC enhancers for *C. robusta, C. savignyi,* and *C. inflata,* we noticed a conserved TGTT-binding motif in both enhancers across all three species (Figs. 3b and Additional file 1: Figure S1B). TGTT is part of the consensus binding motif of Forkhead transcription factors such as FoxF (Additional file 1: Figure S2A) [15]. Prior studies noted the enrichment of this motif in *Cionid* TVC enhancer elements [41] and a recent study also detected a significant enrichment of putative FoxF-binding sites in the predicted *cis*-regulatory elements of a wider range of primary TVC genes [68]. The conservation of this motif suggests that FoxF works to maintain its own expression and activate other primary TVC genes such as *Hand*-*like* in the *C. robusta* cardiopharyngeal GRN. As predicted by this hypothesis, mutation of the TGTT motif (T1) in the minimal *C. robusta Hand*-*like* TVC enhancer (*Cirobu.HL* −1914/−1314::−299) abrogated TVC reporter expression (Additional file 1: Figure S2B). In addition, mutation of the TGTT motif (T1) in the minimal *C. robusta FoxF* TVC enhancer (*Cirobu.FoxF* −1072/−847::pFkh) did not impact TVC expression, as predicted by the hypothetical role of this site in maintaining rather than initiating *FoxF* expression (Additional file 1: Figure S2B). Based on these results, we sought to determine if the TVC enhancer for *GATAa* also contains a conserved TGTT-binding motif. Using our script to computationally predict TVC enhancers for *C. inflata GATAa*, we identified one strong candidate element in the first intron (Additional file 1: Figure S2C), similar to the position of the characterized *C. robusta GATAa* TVC enhancer [61]. A minimal 223 bp region of the intron containing this candidate element fused to a *C. robusta Hand*-*like* minimal promoter (*Coinfl.GATAa* +642/+820::*Cirobu.Hand*-*like* −299) was able to drive reporter expression in the TVCs (Additional file 1: Figure S3). Although the *C. inflata GATAa* enhancer diverged substantially from the *C. robusta* element, it still contains a conserved TGTT-binding motif (Additional file 1: Figure S2C). This finding suggests that *GATAa* is also regulated by *FoxF.* Taken together, these results suggest that FoxF plays a central role in TVC specification, responding rapidly to FGF-dependent Ets1/2 activation, and contributing to the up-regulation of other primary TVC genes including *Hand*-*like,* while also maintaining its own expression. The putative role of *FoxF* upstream of *Hand*-*like* also suggests that the more stringent conservation of the *FoxF*-regulatory element may reflect this more critical functional role.

### Substantial divergence of the *Mesp* cardiopharyngeal founder cell enhancer

**Fig. 5:**
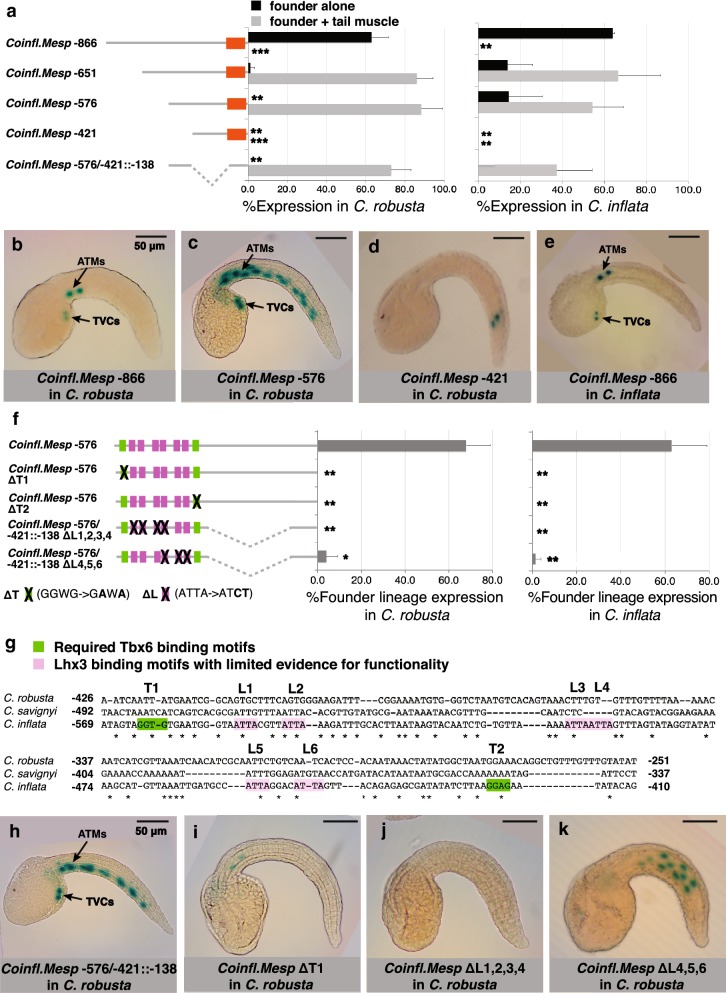
Characterization of the *C. inflata Mesp* founder lineage enhancer. **a** Minimization of the *C. inflata Mesp* 5′ intergenic region to identify the B7.5 founder lineage enhancer. *LacZ* reporter constructs are diagramed on the left. The graphs depict % founder lineage (TVC + ATM) expression or % founder lineage + primary tail muscle lineage expression in *C. robusta* and *C. inflata* (number of trials ≥ 2, total *N* ≥ 25, and error bars indicate standard deviation). Significance relative to *Coinfl.Mesp* −651 was determined with a Student *t* test (*p* < 0.01 indicated by ** and *p* < 0.001 indicated by ***). **b** Representative *C. robusta* embryo showing founder lineage-specific expression of *Coinfl.Mesp* −866 (arrows indicate TVCs and ATMs, and scale bar is 50 μm). **c** Representative *C. robusta* embryo showing the founder lineage and primary tail muscle lineage expression for *Coinfl.Mesp* −576. **d** Representative *C. robusta* embryo showing the lack of founder lineage expression for *Coinfl.Mesp* −421. **e** Representative *C. inflata* embryo showing the founder lineage-specific expression for *Coinfl.Mesp* −866. **f** Effect of TBX6 and LHX3 binding motif knockouts (Δ) on the expression of the *C. inflata Mesp* founder cell enhancer. Binding motifs designated as shown in **g**. *LacZ* reporter constructs are diagramed on the left with an X indicating a binding motif knockout. The graphs depict % founder lineage expression in *C. robusta* and *C. inflata* (number of trials ≥ 2, total *N* ≥ 75, and error bars indicate standard deviation). Significance relative to *Coinfl.Mesp* −576 or the minimal −576/−421 enhancer was determined with a Student *t* test (*p* < 0.05 indicated by * and *p* < 0.01 indicated by **). **g** Structure of the *C. inflata Mesp* founder cell enhancer. Darkly shaded green TBX6 motifs were required for reporter expression, and lightly shaded pink LHX binding motifs exhibited some functionality, as determined by mutagenesis of multiple motifs. There is no conservation of functional binding motifs in the aligned upstream genomic region of *C. robusta*. **h** Representative *C. robusta* embryo showing the founder lineage and primary tail muscle lineage expression for *Coinfl.Mesp* −576/−421::−138. **i**–**k** Representative *C. robusta* embryos showing lack of reporter expression for **i** *Coinfl.Mesp* ΔT1, and **j** *Coinfl.Mesp* ΔL1,2,3,4 and **k** reporter expression in the primary tail muscle lineage, but not the founder lineage for *Coinfl.Mesp* ΔL4,5,6

To further investigate levels of drift across the cardiopharyngeal GRN, we characterized the regulatory element for founder cell expression of *Mesp* in *C. inflata.* In *C. robusta, Mesp is* expressed in the B7.5 cardiopharyngeal founder cell lineage downstream of TBX6b and LHX3 (Fig. 1) [50–53]. Sequence alignments did not reveal a conserved region in *C. inflata* associated with the characterized *Mesp* enhancer in *C. robusta* (Additional file 1: Figure S4A) [66]. We therefore computationally predicted candidate *C. inflata Mesp* enhancers based on binding site clustering. This approach yielded one candidate *cis*-regulatory element that aligned with the known *cis*-regulatory element for *C. robusta* (Additional file 1: Figure S4B) [51]. However, this candidate was a poor match, as it was missing the first two TBX6-binding motifs which were previously shown to be required in *C. robusta* [51]. We therefore started a sequential minimization analysis upstream of the candidate *cis*-regulatory element. The full-length construct (*Coinfl.Mesp* −866) drove strong expression in the founder lineage (ATMs and TVCs) in both *C. inflata* and *C. robusta,* demonstrating mutual intelligibility (Figs. 2, 5a, b, e). This reporter construct displayed almost no background expression (Fig. 5a, b, e). Two shorter constructs (*Coinfl.Mesp* −651 and *Coinfl.Mesp* −576) still drove strong expression in the founder lineage, but also produced ectopic expression in the primary trail muscle lineage (Fig. 5a, c). This result suggests that there is a silencer element 866–576 bp upstream of *Mesp* that represses tail muscle lineage expression. A slightly shorter construct (*Coinfl.Mesp* −421) drove no expression in the founder lineages or primary trail muscle lineages (Fig. 5a, d), indicating that the computationally predicted *cis*-regulatory element was not sufficient for reporter expression. Instead, we found that a region 576–421 bp upstream of *Mesp* fused to a 138 bp basal promoter (*Coinfl.Mesp* −576/−421::−138) drove strong founder lineage expression, demonstrating that this 155 bp region is both necessary and sufficient for founder lineage expression (Fig. 5a). Strikingly, this 155 bp minimal enhancer bears almost no sequence similarity to the characterized *C. robusta* element (Additional file 1: Figure S4C) and is also a very poor match to the globally aligned region 426–261 bp upstream of *C. robusta Mesp* (Fig. 5g) Thus, our analysis reveals substantial divergence between the minimal *Mesp* founder cell enhancers of these two species.

To begin investigating *trans*-regulation of *Mesp* in *C. inflata,* we mutagenized putative binding sites in the minimal reporter construct and assayed the impact on reporter expression in both *C. robusta* and *C. inflata* (Fig. 5f–k). The minimal *C. inflata Mesp* founder cell enhancer contains two TBX6-binding motifs and six LHX3-binding motifs (Fig. 5g). Knocking out either TBX6-binding motif (T1 or T2) completely eliminated founder lineage reporter expression in both *C. robusta* and *C. inflata* (Fig. 5f, i). In contrast, knocking out individual LHX3-binding motifs did not affect founder lineage reporter expression (data not shown). This result could reflect redundancy in the LHX3-binding sites, so we knocked out combinations of LHX3-binding motifs. When we knocked out the first four LHX3-binding motifs (L1, L2, L3, and L4), founder lineage and tail muscle lineage expression were lost in both *C. robusta* and *C. inflata* (Fig. 5f, j). When we knocked out the last three LHX3-binding motifs (L4, L5, and L6), founder lineage expression was almost completely eliminated, but primary tail muscle lineage expression was maintained (Fig. 5f, k). Thus, *trans*-activation of *Mesp* by TBX6 and LHX3 appears to be conserved in *C. inflata* and *C. robusta,* while *cis*-regulatory elements have undergone substantial divergence.

In summary, our data indicate that upstream transcription factors dictating *FoxF, Hand*-*like,* and *Mesp* expression in the cardiopharyngeal GRN are conserved between *C. robusta* and *C. inflata*. However, the *cis*-regulatory elements that control the expression of these genes exhibit distinct levels of conservation between *C. robusta* and *C. inflata.* The *FoxF* TVC enhancer is highly conserved, with identical organization of binding motifs, while the *Hand*-*like* and *Mesp* enhancers exhibit extensive divergence. These distinct levels of *cis*-regulatory conservation do not appear to reflect GRN hierarchy, as Mesp functions at the top of the GRN. Therefore, we began to explore alternative hypotheses regarding the exceptional conservation of the *FoxF* TVC enhancer over ~ 270 million years of rapid evolutionary divergence.

### Precise binding site spacing is required for *FoxF* TVC enhancer function

**Fig. 6:**
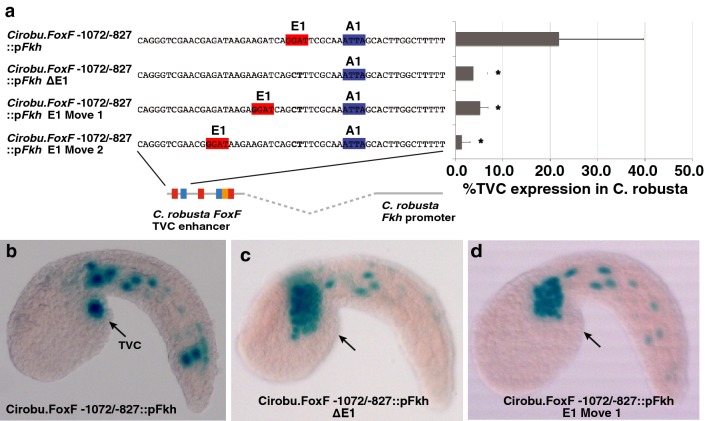
Functional constraint on binding site spacing in the *C. robusta FoxF* TVC enhancer. **a** The first Ets1/2-binding site was moved by knocking out the endogenous binding site (GGAT ⟶ GCTT) and introducing a new binding site using site-directed mutagenesis. Reporter constructs contained the 245 bp minimal *C. robusta FoxF* TVC enhancer fused to the *C. robusta Fkh* basal promoter (*Cirobu.FoxF* −1072/−827::p*Fkh*). The sequence of the enhancer region containing this first Ets1/2-binding site is shown on the left with Ets1/2 (red) and ATTA (blue)-binding sites highlighted. The graph depicts %TVC expression in *C. robusta* (number of trials ≥ 2, total *N* ≥ 75, and error bars indicate standard deviation). **b**–**d** Representative *C. robusta* embryos showing reporter expression for **b** *Corobu.FoxF* −1072/−827::pFkh, **c** *Corobu.FoxF* −1072/−827::pFkh ∆E1, or **d** *Corobu.FoxF* −1072/−827::pFkh Move 1. Arrows point to normal position of TVCs in the trunk region. Note substantial ectopic expression in the anterior tail muscle lineage (ATM) and in other muscle and mesenchyme lineage cells

There are a number of possible explanations for the relatively stringent conservation of the *FoxF* TVC enhancer between *C. inflata* and *C. robusta*. The first is that a specific organization of binding sites is required for physical interactions between transcription factors [3, 14]. Alternatively, the enhancer may be constrained to ensure precise temporal or spatial expression [69]. To distinguish between these hypotheses, we displaced the first Ets1/2-binding motif (E1) in the *C. robusta FoxF* TVC enhancer and examined the impact on reporter expression. We chose this binding site because it is required for strong TVC expression in both *C. robusta* and *C. inflata* (Fig. 3b, g). Moreover, the ten base-pair spacing between this binding motif (E1) and the first ATTA-binding motif (A1) is conserved between *C. robusta* and *C. inflata.* A ten base-pair increment between binding sites corresponds to a single helical turn and is often observed in enhanceosome-like *cis*-regulatory elements [14]. We displaced this first Ets1/2-binding site by knocking out the endogenous site and introducing a new site either 16 or 24 base pairs from the first ATTA site. We conducted this analysis in a *LacZ* reporter construct containing the minimal 245 bp *C. robusta FoxF* TVC enhancer fused to the basal Forkhead promoter (*Cirobu.FoxF* −1072/−827::*pFkh:lacZ*). This is a slightly longer construct than the previously characterized 232 bp minimal reporter (*Cirobu.FoxF* −1072/−840::*pFkh:lacZ*) [58]. When the first Ets1/2-binding motif (E1) was knocked out in the context of the 245 bp minimal element, TVC reporter expression was significantly reduced (Fig. 6a, c). The introduction of new Ets1/2-binding sites 6 bp (Move 1), or 14 bp (Move 2) upstream of the original position failed to rescue TVC reporter expression (Fig. 6a, d). The fact that this reorganization reduced expression rather than altering temporal or spatial expression patterns supports the hypothesis that binding site organization is constrained by required interactions between *trans*-factors.

## Discussion

### Developmental systems drift within the tunicate cardiopharyngeal GRN

Mutual intelligibility in our cross-species assays suggests that the *trans*-regulatory architecture of the cardiopharyngeal GRN is largely conserved between *C. inflata* and *C. robusta*. These findings are in contrast to previous comparisons between *M. occidentalis* and *C. robusta* that revealed numerous instances of enhancer incompatibility caused by extensive *trans* drift in the cardiopharyngeal GRN [10]. Both these studies are based on functional analysis of minimal regulatory elements and thus may not encompass the full range of cis-regulatory function (as mentioned in the introduction, our use of the term drift in this instance and throughout the discussion is speculative, because observed changes in GRN structure may have undetected impacts on expression and thus may not be independent of selection). However, these studies still provide a robust framework for developing models regarding the rate and nature of developmental systems drift. In particular, these findings are congruent with two alternative models for the emergence of *trans* drift in developmental GRNs. *Trans* drift may arise at a steady rate, so that the amount of drift roughly correlates with the absolute evolutionary distance between two species and is not influenced by other taxonomic considerations. Alternatively, the rate of *trans* drift may vary due to factors independent of evolutionary distance. In particular, increased drift may occur during the divergence of major clades, such as that between phlebobranchs and stolidobranchs, in association with shifts in morphology or rewiring of underlying developmental gene networks. According to the first model, the differential occurrence of *trans* drift between *M. occidentalis* and *C. robusta* can be attributed to the longer period of divergence between these species*, *~ 390 million years, in comparison with *C. inflata,* which diverged from *C. robusta *~ 270 million years ago [43]. According to the second model, differential *trans* drift may have arisen during GRN rewiring associated with changes in body plan or divergence of developmental programs between *Phlebobranchs* and *Stolidobranchs*. A broader cross-species analysis is required to distinguish between these models.

Our analysis of the *Mesp* founder cell enhancer also provides an alternative perspective on differential divergence between *trans*-regulatory inputs [70]. The activation of *Mesp* by TBX6b is conserved between *M. occidentalis, C. inflata,* and *C. robusta*, while its activation by LHX3 is only conserved between *C. inflata* and *C. robusta*. Our results suggest that differential levels of constraint on these *trans*-factor inputs reflect a primary directive role for TBX6b, while LHX3 plays a more secondary, permissive role. When we removed the 300 bp genomic region upstream of the *C. inflata Mesp* founder cell enhancer, we observed ectopic primary tail muscle lineage reporter expression. A similar result has been observed during deletion analysis of the *C. robusta Mesp* enhancer (Brad Davidson, unpublished results). Ectopic tail muscle expression is likely caused by TBX6b, which is expressed in a broad domain encompassing the B7.5 founder cells and neighboring tail muscle lineages [53]. According to this model, regions’ upstream of the minimal *Mesp* element may contain a silencer bound by a tail muscle specific repressor. Thus, in tail muscle lineages, TBX6 may be able to activate *Mesp* expression independently of LHX3, which is expressed only in the endoderm/founder lineage cells. We are unsure why one set of LHX3 binding motif knockouts eliminated primary tail muscle and founder lineage expression, while another set only eliminated founder lineage expression. It is possible that mutagenesis of the first four LHX3-binding motifs accidentally impacted the binding motif of an additional transcription factor required for *Mesp* activation. Overall, our results provide preliminary support for the hypothesis that heterogeneous levels of constraint on *trans*-regulatory inputs reflect directive rather than permissive functional contributions. Clearly, further analysis is required to solidify our understanding of *Mesp* regulation and further test this general hypothesis.

Our findings provide more robust insights into *cis*-regulatory drift. Sequence alignments and functional enhancer analysis reveal highly variable levels of divergence for *cis*-regulatory elements within the cardiopharyngeal GRN. The minimal *FoxF* TVC enhancer is highly conserved, with identical organization and spacing of binding motifs. In contrast, the minimal *Hand*-*like* TVC enhancer is poorly conserved and the minimal *Mesp* founder cell lacks any apparent structural conservation. These findings do not align with models in which differential constraints associated with the position or function of a gene in a GRN dictate relative levels of *cis*-regulatory drift. Rather, our findings suggest that drift is dictated by distinct structural and functional constraints that are unique to each *cis*-regulatory element. Our findings have also begun to illuminate the specific structural and functional constraints that dictate conservation of the *FoxF* enhancer, as discussed in the following section.

### Model for the constraints on the *FoxF* TVC enhancer

Highly conserved enhancers generally reflect cooperative, position-specific interactions between bound transcription factors [14]. This type of highly conserved enhancer is known as an enhanceosome and is distinguished by conservation of the number, order, orientation, and spacing of binding motifs [3, 14]. The prototypical enhanceosome is the interferon-β *cis*-regulatory element [71]. Although relatively rare, additional enhanceosome-like *cis*-regulatory elements have subsequently been characterized [14, 17–19, 72]. However, general principles regarding the deployment of enhanceosomes within developmental GRNs have not been delineated. Mutations that disrupt the relative position of binding sites generally disable enhanceosome elements, presumably because they disrupt protein–protein interactions [16]. We show that displacing the first Ets1/2-binding motif in the *C. robusta FoxF* TVC enhancer significantly reduces reporter expression. This result suggests that the *FoxF* TVC enhancer is an enhanceosome-like *cis*-regulatory element, in which Ets1/2, the ATTA-binding co-factor, and possibly other proteins must physically interact to activate *FoxF* expression. However, further experimentation will be required to provide more definitive support for this hypothesis. In particular, the use of a wider range of mutations will help determine whether the specific mutations we introduced had unintended impacts, such as the creation or elimination of cryptic binding sites. In addition, by further varying binding site displacement, we can test whether presumed cooperativity is dependent on relative position on the helix. Furthermore, it will be interesting to analyze whether the conserved distances between other binding motifs in the *FoxF* minimal enhance also reflect functional constraints.

The deployment of an enhanceosome for regulation of *FoxF* may be associated with its role as a pioneer factor. This hypothesis arises from the recent findings of Racioppi et al., who found that FoxF promotes TVC specification by changing chromatin accessibility [68]. In particular, the binding of FoxF to the enhancers of other early TVC genes, including *Hand*-*like* and *GATAa*, appears to increase the accessibility of these *cis*-regulatory elements by decondensing chromatin, thereby enabling activation of these genes by Ets1/2, and the ATTA-binding co-factor [68]. Racioppi et al. also showed that CRISPR/Cas9 knockdown of *FoxF* led to down-regulation of several early TVC genes, including *Hand*-*like* [68]. Our mutational analysis of the FoxF-binding motif in the *C. robusta Hand*-*like* and *FoxF* TVC enhancer further supports the hypothesis that FoxF acts as a pioneer factor during TVC specification and also suggests that FoxF maintains its own expression.

## Conclusion

**Fig. 7:**
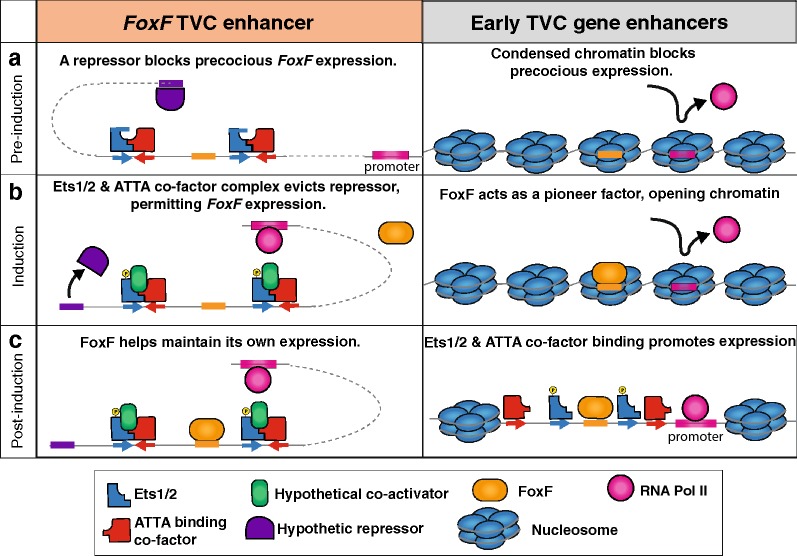
Model for the differential constraint on *FoxF* vs. other early TVC enhancers. **a** Before FGF induction, the chromatin around early TVC gene enhancers is condensed preventing aberrant expression. In contrast, chromatin is decondensed at the *FoxF* TVC enhancer locus, suggesting that a repressor (purple) is required to prevent precocious expression. **b** FGF/MapK-signaling phosphorylates Ets1/2 in the TVCs, permitting recruitment of a co-factor (green) that serves to lift repression. The cooperative recruitment of this co-factor constrains binding site position and orientation. FoxF (orange) then accumulates in the TVC nuclei, where it acts as a pioneer factor opening the chromatin around other TVC enhancers. **c** Once early TVC gene enhancers are open, the binding of Ets1/2, ATTA, and FoxF activates transcription in a non-cooperative fashion, as reflected by a lack of constraint on binding site position. FoxF also binds the *FoxF* TVC enhancer helping to maintain its own expression

Taken together, these results allow us to formulate a model that explains the specific deployment of a highly constrained, enhanceosome-like element for the regulation of *FoxF* (Fig. 7). Before FGF induction, the chromatin around the enhancers of most early TVC genes is condensed, which prevents aberrant expression (Fig. 7a). One exception is the *FoxF* enhancer, which remains decondensed, so it can mediate a rapid, primary response to FGF/MapK-dependent activation of Ets1/2 (Fig. 7a). Since chromatin condensation does not constrain aberrant expression of *FoxF,* another mechanism is required. We propose that this alternate mechanism involves the occupation of a silencer element located near the FoxF enhancer. Indeed, ectopic reporter expression throughout the B7.5 founder lineage in our 245 bp minimal FoxF enhancer construct (Fig. 6b) suggests that a silencer element serves to block precocious FoxF expression, possibly mediated by unphosphorylated Ets. According to our model, FGF/MapK-dependent phosphorylation of Ets1/2 leads to the formation of a complex with the ATTA-binding factor and the recruitment of a presumptive, non-DNA binding co-factor that is able to lift baseline repression (Fig. 7b). Once the *FoxF* gene is expressed, FoxF maintains its own expression and opens the chromatin around other TVC enhancers (Fig. 7c). This model may reflect a general principle for the seemingly sporadic occurrence of enhanceosomes. Namely, enhanceosomes may be specifically deployed for pioneer *trans*-factors, ensuring precise temporal or spatial expression despite a lack of chromatin-dependent regulation.

## Methods

### Computational enhancer prediction

The enhancers for *C. inflata Hand*-*like, GATAa,* and *Mesp* were computationally predicted based on structural similarity to the previously characterized enhancers in *C. robusta* [50, 51, 61]. A custom Python (version 2.7.13) script was used to slide a 150 bp window over the *C. inflata* 5′ intergenomic region for each of these genes in 25 bp increments (https://github.com/colganwi/CRMFinder). Each window position was scored with a linear combination of four features [1]: the number of oligomers ≥ 4 bp which were present in both the window and the *C. robusta* enhancer, allowing for reverse complements, [2] similarity in oligomer ordering—the number of steps needed to transform one ordering into the other normalized by the number of conserved oligomers [3], similarity in enhancer position—the difference in the distance to the start codon normalized by the size of the 5′ intergenic region, and [4] the presence of specific conserved motifs, Ets1/2 (GGAW) for *Hand*-*like* and *GATAa* and TBX6 (GGNG) for *Mesp*.

### Molecular cloning

#### *LacZ* reporter constructs

Molecular cloning was performed according to established protocols [51]. *C. inflata* genomic regions used for enhancer analysis were amplified with sequence-specific primers carrying appropriate restriction sites (Additional file 1: Table S1). Cloning of *C. robusta FoxF* and *Hand*-*like* minimal enhancers was described by Beh et al. and Woznica et al. [41, 58].

#### Site-directed mutagenesis or insertion

Sequence-specific primers containing desired point mutations or insertions (Additional file 1: Table S2) were used to generate sticky end fragment [51] or for whole plasmid amplification. For single-step whole plasmid amplification, we used mutagenesis primers between 30 and 60 bases in length, with a melting temperature (Tm) of ≥ 78 °C, the mutation placed in the exact center of the primer with 10–30 bp of correct sequence on both sides, and a minimum GC content of 40%. Primers were diluted to 125 ng/μl and PCR run with 5–50 ng of template, Pfu ultra II taq polymerase (Agilent). If template was > 5 kb, we added 3 μl DMSO, and the reaction was run for 12–30 cycles based on the extent of the mutagenesis (12 for point mutations, 16 for 2–3 bp mutations, up to 30 for larger mutations). The PCR reaction was then cut with 1–2 μl of *Dpn*I at 37 °C for 1 h and incubated at 70 °C for 20 min prior to transformation of competent cells according to standard protocols.

### Embryological techniques

#### Fertilization and dechorionation

Adult *C. inflata* were harvested from docks on Lopez or San Juan Island, WA. M_REP (Carlsbad, CA) supplied adult *C. robusta* from multiple collection locations along the coast of San Diego, CA. *C. robusta* fertilization, dechorionation, electroporation, and staging were carried out as previously described [30, 56, 73]. For *C. inflata,* similar protocols were used with the following modifications. Sperm and then eggs were dissected from 4 to 6 gravid, freshly collected adults. Concentrated sperm from all adults was mixed in a 10 ml dish of FNSW (filtered natural sea water). Eggs were dissected from each individual into a separate small dish of FNSW, and then, all eggs were rinsed once using 70 μm mesh. Sperm was added to rinsed eggs, and after 12 min, zygotes were passed through six rinse dishes. The zygotes were then transferred to a 10 ml dish, and excess water was removed and replaced with a dechorionation solution (10 ml FNSW + a 200 μl freshly thawed aliquot of 5% protease in FSW *Streptomyces griseus*, Sigma P8811-1G). After 4 min, zygotes were pipetted gently and checked for dechorionation every minute. After ~ 9–11 min, dechorionated zygotes were rinsed sequentially in six 10 ml dishes of FNSW. Electroporation was as described for *C. robusta* except that only 50 μl of total mannitol + DNA solution was used. Embryos were transfected with 100–300 μg of DNA. Higher time constants (~ 20 ms) appeared to give the best incorporation and did not hinder development. Embryos were cultured in gelatin-coated dishes with 10 ml of FNSW on a floating platform in a sea table (~ 14–16 °C) with the lids upside down to ensure that sea table water did not enter the cultures. Embryos were transferred after 2–4 h (4–16 cell stage) to a fresh dish of FNSW to ensure proper development.

#### X-gal staining

Stage 22–23 embryos were fixed with 0.175% glutaraldehyde and then stained with X-gal to visualize *LacZ* reporter expression as previously described [51].

## Supplementary information

**Additional file 1.** Additional figures and tables.

## Data Availability

All data, scripts, sequences, and plasmid constructs will be made publicly available once the manuscript is accepted for publication.

## Abbreviations

GRNs: gene regulatory networks
TVCs: trunk ventral cells
FGF: fibroblast growth factor
MapK: Map Kinase
ATM: anterior tail muscle
