# Supplementary material for: Variable levels of drift in tunicate cardiopharyngeal gene regulatory elements
Source: EvoDevo. 2019 Oct 11;10:24. doi: 10.1186/s13227-019-0137-2 (PMC6790052; doi:10.1186/s13227-019-0137-2)
Supplement: Supplementary file 1 — Additional file 1. Additional figures and tables. [file 13227_2019_137_MOESM1_ESM.docx]

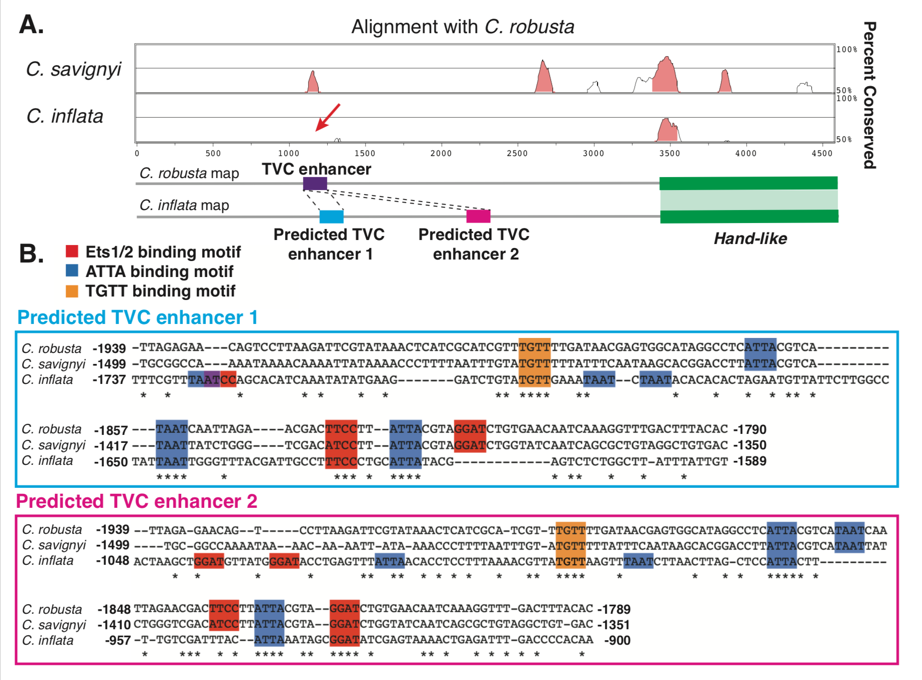


**Figure S1: Candidate *C. inflata* *Hand-like* regulatory elements.** (**A**) mVISTA alignments depict sequence conservation between *C. robusta* and *C. savignyi* and between *C. robusta* and *C. inflata* for the *Hand-like* gene and 5’ intergenic region (LAGAN alignment, conservation across 100bp window, conservation > 70% highlighted). There is increased conservation between the two Cionid species associated with the *Hand-like* gene (green) and *Ciona* TVC enhancer (purple), but this enhancer is not conserved in *C. inflata* (red arrow). (**B**) Computational prediction of TVC enhancers for *C. inflata Hand-like* based on clustering of conserved binding motifs. The first prediction (cyan) is 1737bp upstream of the gene and the second prediction (pink) is 957bp upstream of the gene. ClustalW alignment (default settings) of the two predicted enhancers with the 149bp *Ciona* enhancer reveals limited structural similarities associated with the presence of Ets or ATTA binding motifs.


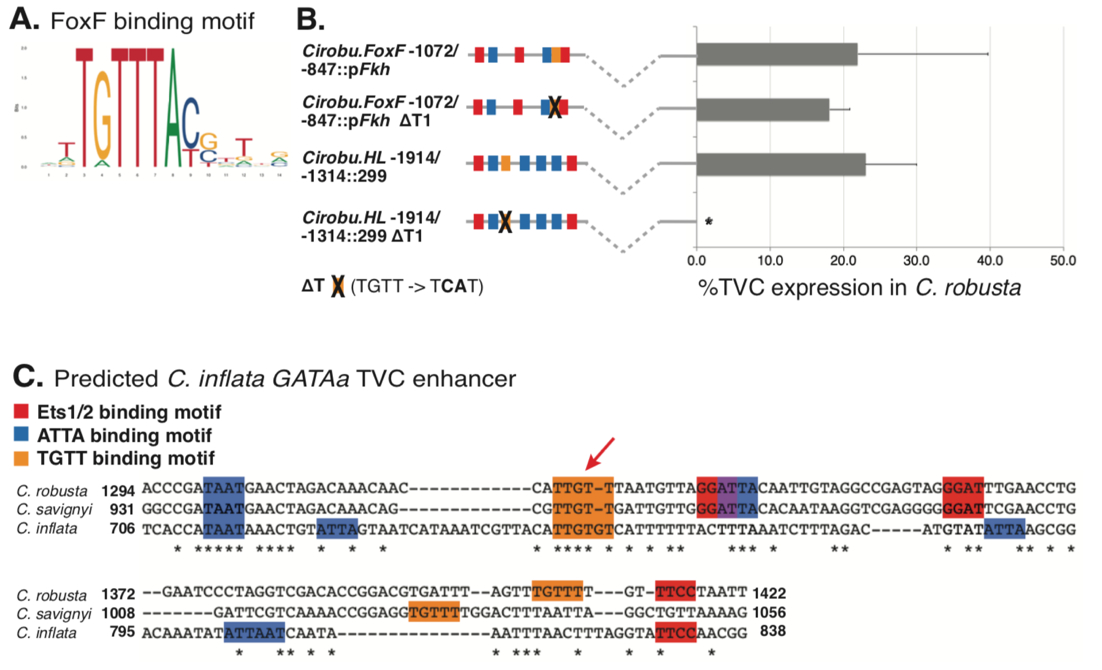


**Figure S2: Characterization of a presumptive FoxF binding site in early TVC enhancers.** (**A**) The vertebrate FoxF binding motif from JASPAR (15). This motif is conserved in both the *FoxF* and *Hand-like* TVC enhancers (see Figures 3B and Supplemental Figure 1B). (**B**) Effect of TGTT binding motif knockouts (Δ) on the expression of the *C. robusta* TVC enhancers for *FoxF* (*Cirobu.FoxF* -1072/-847::p*Fkh*) and *Hand-like* (*Cirobu.HL* -1914/-1314::299). The graph depicts %TVC expression in *C. robusta* (number of trials ≥ 2, total N ≥ 75, error bars indicate standard deviation). Significance for both graphs was determined with a Student t-test (p<0.05 indicated by *). (**C**) The computationally predicated TVC enhancer for *C. inflata GATAa* also contains a TGTT binding motif which is conserved in *C. robusta* (red arrow).


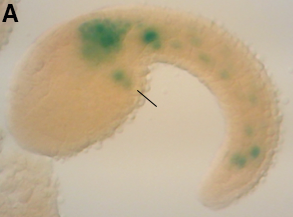


**Figure S3: An intronic enhancer for *C. inflata GATAa*** (**A**) A minimal *C. inflata* intronic enhancer drives reporter expression in the TVCs (line) when placed upstream of a minimal *C. robusta* *Hand-like* promoter (*Coinfl.GATAa* +642/+820::*cirobuHand-like* 299::*lacZ*) and electroporated into *C. robusta* embryos.


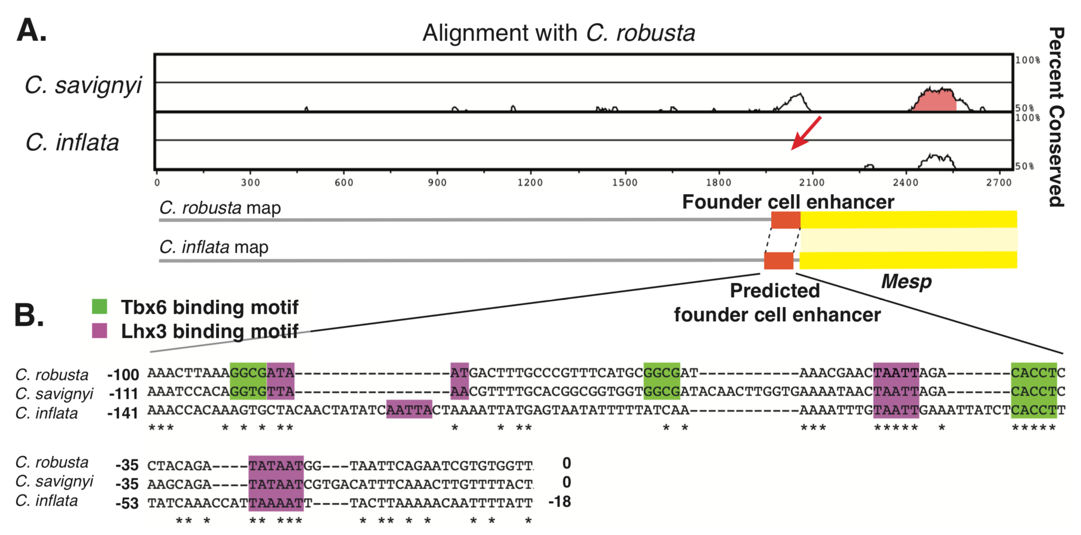


**C.
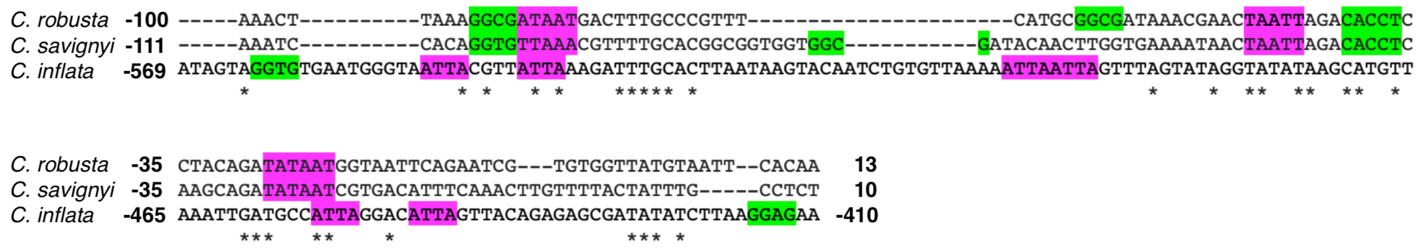
**

**Figure S4: Identification of a candidate *C. inflata* *Mesp* founder cell enhancer.** (**A**) mVISTA alignments depict sequence conservation between *C. robusta* and *C. savignyi* and between *C. robusta* and *C. inflata* for the *Mesp* gene and 5’ intergenic region (LAGAN alignment, conservation across 100bp window, conservation > 70% highlighted). There is increased conservation associated with the *Mesp* gene (yellow) and *Ciona* Founder cell enhancer (orange) in the two Cionid species, but this region is not highly conserved in *C. inflata* (red arrow). (**B**) ClustalW alignment of the 100bp *C. robusta* founder enhancer with the orthologous region in *C. inflata* (default settings). Tbx6 (green) and Lhx3 (purple) binding motifs are highlighted. (**C**) ClustalW alignment of the 100bp *C. robusta* founder enhancer with the characterized minimal element in *C. inflata* (default settings). Tbx6 (green) and Lhx3 (purple) binding motifs are highlighted.

**Table S1: Primers for cloning of candidate regulatory elements**

| Gene | Strand | Position | Restriction site | Sequence |
| --- | --- | --- | --- | --- |
| *Coinfl.Foxf* | + | -2622 | Pst1 | aaaCTGCAGTCGTATACAGGCTAGCTGCGG |
| *Coinfl.Foxf* | + | -547 | Pst1 | aaaCTGCAGATCGAAGCCAGGAATTATAAATTAGCACCTGGC |
| *Coinfl.Foxf* | - | -401 | Xba1 | aaaTCTAGAGTACGGAAACAATTACATAAAACGG |
| *Coinfl.Foxf* | - | +9 | Not1 | aaaGCGGCCGCTGAAACTTCCATTCTGG |
| *Coinfl.HL* | + | -1737 | Xba1 | aaaTCTAGATTCGTTTAATCCAGCACATC |
| *Coinfl.HL* | + | -1615 | Xba1 | aaaTCTAGACGAGTCTCTGGCTTATTTATTG |
| *Coinfl.HL* | + | -1048 | Xba1 | aaaTCTAGATAGGACCTTGTGGAATTTGG |
| *Coinfl.HL* | + | -899 | Xba1 | aaaTCTAGACATTAAATAGCGGATATCGAG |
| *Coinfl.HL* | *-* | -844 | Not1 | aaaGGATCCGTCACAGCCTAAATCTCAGT |
| *Coinfl.HL* | *-* | +1 | Not1 | aaaGCGGCCGTATCAGAGAGGGTCATTTTGCAAG |
| *Coinfl.Mesp* | + | -866 | Xba1 | aaaTCTAGACAACATGATCACTAAAC |
| *Coinfl.Mesp* | + | -651 | Xba1 | aaaTCTAGACATCTAAAGCCAAAAGGTG |
| *Coinfl.Mesp* | + | -576 | Xba1 | aaaTCTAGACATAGTAGGTGTGAATGG |
| *Coinfl.Mesp* | + | -421 | Xba1 | aaaTCTAGAGGAGAATATACAGGAAAAGG |
| *Coinfl.Mesp* | - | -421 | BamH1 | aaaGGATCCCCTTTTCCTGTATATTCTCC |
| *Coinfl.Mesp* | - | +1 | Not1 | aaaGCGGCCGCATCAGGCAGGTGTTGCCTTAAG |

**Table S2: Mutagenesis Primers**

| Gene | Strand | Mutation | Sequence |
| --- | --- | --- | --- |
| *Coinfl.FoxF* | + | ΔA1  (ATTA -> AA**CT**) | aaaCTGCAGATCGAAGCCAGGAATTATAAATACGCACCTGGC |
| *Coinfl.FoxF* | + | ΔA2  (ATTA -> AA**CT**) | aaaTCTAGAGTACGGAAACAATACCATAAAACGG |
| *Coinfl.FoxF* | + | ΔE1  (GGAW -> **TAG**W) | aaaCTGCAGATCGAAGCCATAGATTATAAATTAGCACCTGGC |
| *Coinfl.FoxF* | + | ΔE2  (GGAW -> **TAG**W) | AGCACCTGGCTTGCTGTGGCCTGATAAATGTATCCTATACGGTA GCTAAGTAGTCAC |
| *Coinfl.FoxF* | - | ΔE2  (GGAW -> **TAG**W) | GTGACTACTTAGCTACCGTATAGGATACATTTATCAGGCCACAG CAAGCCAGGTGCT |
| *Coinfl.FoxF* | - | ΔE3  (GGAW -> **TAG**W) | aaaTCTAGAGTACTAGAACAATTACATAAAACGG |
| *Coinfl.HL* | + | ΔA1  (ATTA -> AA**CT**) | GTTATGGGATACCTGAGTTTATCTACACCTCCTTTAAAACGTTATG |
| *Coinfl.HL* | - | ΔA1  (ATTA -> AA**CT**) | CATAACGTTTTAAAGGAGGTGTAGATAAACTCAGGTATCCCATAAC |
| *Coinfl.HL* | + | ΔA2  (ATTA -> AA**CT**) | CCTTTAAAACGTTATGTTAAGTTAGATCTTAACTTAGCTCCATTACTT |
| *Coinfl.HL* | - | ΔA2  (ATTA -> AA**CT**) | AAGTAATGGAGCTAAGTTAAGATCTAACTTAACATAACGTTTTAAAGG |
| *Coinfl.HL* | + | ΔA3  (ATTA -> AA**CT**) | AAGTTTAATCTTAACTTAGCTCCATCTCTTTTGTCGATTTACATTAAATAGC |
| *Coinfl.HL* | - | ΔA3  (ATTA -> AA**CT**) | GCTATTTAATGTAAATCGACAAAAGAGATGGAGCTAAGTTAAGATTAAACTT |
| *Coinfl.HL* | + | ΔA4  (ATTA -> AA**CT**) | CCATTACTTTTGTCGATTTACATACAATAGCGGATATCGAGTAAAA |
| *Coinfl.HL* | - | ΔA4  (ATTA -> AA**CT**) | TTTTACTCGATATCCGCTATTGTATGTAAATCGACAAAAGTAATGG |
| *Coinfl.HL* | + | ΔE1  (GGAW -> G**CT**W) | ATTTTAACTAAGCTGGATGTTATGGCTTACCTGAGTTTATTAACACCTCC |
| *Coinfl.HL* | - | ΔE1  (GGAW -> G**CT**W) | GGAGGTGTTAATAAACTCAGGTAAGCCATAACATCCAGCTTAGTTAAAAT |
| *Coinfl.HL* | + | ΔE2  (GGAW -> G**CT**W) | TTTGTCGATTTACATTAAATAGCGCTTATCGAGTAAAACTGAGATTTAGG |
| *Coinfl.HL* | - | ΔE2  (GGAW -> G**CT**W) | CCTAAATCTCAGTTTTACTCGATAAGCGCTATTTAATGTAAATCGACAAA |
| *Coinfl.Mesp* | + | ΔL1  (ATTA -> AA**CT**) | AGTAGGTGTGAATGGGTAtcTACGTTATTAAAGATTTG |
| *Coinfl.Mesp* | - | ΔL1  (ATTA -> AA**CT**) | CAAATCTTTAATAACGTAgaTACCCATTCACACCTACT |
| *Coinfl.Mesp* | + | ΔL2  (ATTA -> AA**CT**) | GTGAATGGGTAATTACGTTATctAAGATTTGCACTTAATAAGTG |
| *Coinfl.Mesp* | - | ΔL2  (ATTA -> AA**CT**) | CACTTATTAAGTGCAAATCTTagATAACGTAATTACCCATTCAC |
| *Coinfl.Mesp* | + | ΔL3  (ATTA -> AA**CT**) | GTTATTAAAGATTTGCACTagATAAGTGCAATCTGTGTTAAA |
| *Coinfl.Mesp* | - | ΔL3  (ATTA -> AA**CT**) | TTTAACACAGATTGCACTTATctAGTGCAAATCTTTAATAAC |
| *Coinfl.Mesp* | + | ΔL4  (ATTA -> AA**CT**) | TAAGTGCAATCTGTGTTAAAAATctcTTAGTTTAGTATAGGTATATAAGC |
| *Coinfl.Mesp* | - | ΔL4  (ATTA -> AA**CT**) | GCTTATATACCTATACTAAACTAAgagATTTTTAACACAGATTGCACTTA |
| *Coinfl.Mesp* | + | ΔL5  (ATTA -> AA**CT**) | GCATGTTAAATTGATGCCATctGGACATTAGTTACAGAGAGC |
| *Coinfl.Mesp* | - | ΔL5  (ATTA -> AA**CT**) | GCTCTCTGTAACTAATGTCCagATGGCATCAATTTAACATGC |
| *Coinfl.Mesp* | + | ΔL6  (ATTA -> AA**CT**) | AATTGATGCCATTAGGACATctGTTACAGAGAGCGATATATC |
| *Coinfl.Mesp* | - | ΔL6  (ATTA -> AA**CT**) | GATATATCGCTCTCTGTAACagATGTCCTAATGGCATCAATT |
| *Coinfl.Mesp* | + | ΔT1  (GGWG -> G**A**G**A**) | GGTCGACTCTAGACATAGTAGaTaTGAATGGGTAATTACGTTAT |
| *Coinfl.Mesp* | - | ΔT1  (GGWG -> G**A**G**A**) | ATAACGTAATTACCCATTCAtAtCTACTATGTCTAGAGTCGACC |
| *Coinfl.Mesp* | + | ΔT2  (GGWG -> G**A**G**A**) | CAGAGAGCGATATATCTTAAGaAaAATATACAGGAAAAGGTAAT |
| *Coinfl.Mesp* | - | ΔT2  (GGWG -> G**A**G**A**) | ATTACCTTTTCCTGTATATTtTtCTTAAGATATATCGCTCTCTG |
| *Coinfl.Mesp* | + | ΔL1,2,3  (ATTA -> AA**CT**) | GGTGTGAATGGGTAtcTACGTTATctAAGATTTGCACTagATAAGTGCAATCTGTGTTA |
| *Coinfl.Mesp* | - | ΔL1,2,3  (ATTA -> AA**CT**) | TAACACAGATTGCACTTATctAGTGCAAATCTTagATAACGTAgaTACCCATTCACACC |
| *Coinfl.Mesp* | + | ΔL5,6  (ATTA -> AA**CT**) | GCATGTTAAATTGATGCCATctGGACATctGTTACAGAGAGCGATATATC |
| *Coinfl.Mesp* | - | ΔL5,6  (ATTA -> AA**CT**) | GATATATCGCTCTCTGTAACagATGTCCagATGGCATCAATTTAACATGC |
| *Cirobu.FoxF* | + | ΔT1  (TGTT -> T**AC**T) | CCAGCGTGGACGAAAAGGTAATTcaTTCCGAATGGATCGCGTTCGAGA |
| *Cirobu.FoxF* | - | ΔT1  (TGTT -> T**AC**T) | TCTCGAACGCGATCCATTCGGAAtgAATTACCTTTTCGTCCACGCTGG |
| *Cirobu.FoxF* | + | ΔE  (GGAW -> G**CT**W) | GAACGAGATAAGAAGATCAGctTTCGCAAATTAGCACTTGGC |
| *Cirobu.FoxF* | - | ΔE1  (GGAW -> G**CT**W) | GCCAAGTGCTAATTTGCGAAagCTGATCTTCTTATCTCGTTC |
| *Cirobu.FoxF* | + | E1 Move 1 | CAGGGTCGAACGAGATAAGAgGATCAGctTTCGCAAATTAGCACTTGGC |
| *Cirobu.FoxF* | - | E1 Move 1 | GCCAAGTGCTAATTTGCGAAagCTGATCcTCTTATCTCGTTCGACCCTG |
| *Cirobu.FoxF* | + | E1 Move 2 | GCTTACTAGAATCAGGGTCGAACGgGATAAGAAGATCAGctTTCGCAAATTAGCACTTGG |
| *Cirobu.FoxF* | - | E1 Move 2 | CCAAGTGCTAATTTGCGAAagCTGATCTTCTTATCcCGTTCGACCCTGATTCTAGTAAGC |
| *Cirobu.HL* | + | ΔT1  (TGTT -> T**AC**T) | CGTATAAACTCATCGCATCGTTTacTTTGATAACGAGTGGCATAGGCC |
| *Cirobu.HL* | - | ΔT1  (TGTT -> T**AC**T) | GGCCTATGCCACTCGTTATCAAAgtAAACGATGCGATGAGTTTATACG |
